# An Unusual Presentation of Failure to Thrive in a Toddler: Bartter Syndrome

**DOI:** 10.7759/cureus.67289

**Published:** 2024-08-20

**Authors:** Akshai R, Sakshi Upendra Bhatia, Kishore Narayan, Syed Mohammed, Pallavi Yelkur

**Affiliations:** 1 Paediatrics, Saveetha Medical College and Hospital, Saveetha Institute of Medical and Technical Sciences, Saveetha University, Chennai, IND

**Keywords:** polyhydramnios, dehydration, hypokalemia, metabolic alkalosis, bartter syndrome

## Abstract

Bartter syndrome is a rare salt-wasting renal tubular disorder of autosomal-recessive inheritance. Antenatal Bartter syndrome (types I, II, and IV) manifests in infancy and has a more severe course compared to the classic Bartter syndrome (type III). This report details a unique instance of a male toddler, aged 18 months, who presented with failure to thrive, polydipsia, and polyuria. Blood gases revealed hypochloremic metabolic alkalosis with hyponatremia and hypokalemia. The diagnosis was confirmed by genetic testing, and the child was started on indomethacin and potassium supplementation. Despite being rare in children, this case report emphasizes the importance of looking beyond the usual in a child who presents with failure to thrive to prevent a delay in the diagnosis and treatment.

## Introduction

The term Bartter syndrome encompasses a group of diseases, which include antenatal Bartter syndrome (types I, II, and IV) that is usually present in infancy, classic Bartter syndrome (type III), and Gitelman syndrome. Antenatal Bartter syndrome includes maternal polyhydramnios, neonatal salt wasting, and recurrent dehydration. It is also called hyperprostaglandin E syndrome. The classic form of the disease is milder and usually presents with polyuria, polydipsia, and failure to thrive, which usually begins in the first two years of life [1]. It is characterized by the dysfunction of one of the transporters responsible for sodium chloride reabsorption in the thick ascending limb of the loop of Henle. This transporter can be the apical potassium channel called the renal outer medullary potassium channel, the basolateral chloride channel, the sodium-potassium-2 chloride cotransporter (NKCC 2), or the combined chloride channel [2].

The classical biochemical abnormalities include hypokalemic hyperchloremic metabolic alkalosis, along with hypercalciuria and wasting of salt. This rare genetic renal tubular disease is characterized by hyperreninemia, normal blood pressure, and increased urinary losses of sodium, potassium, calcium, and chloride [3]. Clinical similarities trigger one to think in line with malnutrition or chronic conditions like cystic fibrosis, resulting in a prolonged list of investigations and loss of time in managing the case. The rarity of genetic conditions like Bartter syndrome makes it even more difficult for the clinician to consider it in the first instance. 

It may be even more confusing, especially in conditions like chronic heart failure or chronic kidney disease, when children will be under diuretics, and the presentation attributed to its long-term use. Here, we present a case of Bartter syndrome, which missed attention till 1.5 years of age.

## Case presentation

**Figure 1:**
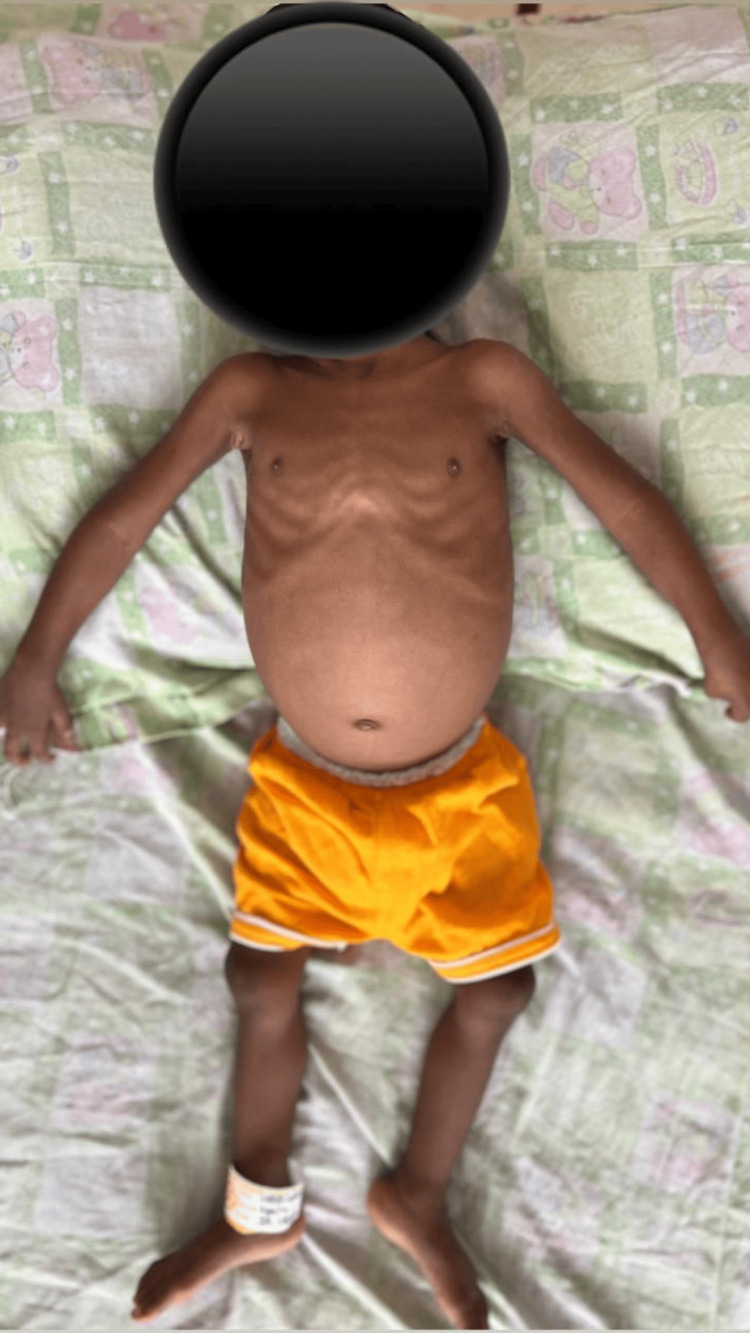
Child with signs of malnutrition at the time of admission The image shows a malnourished child with a wasting, protuberant abdomen.

An 18-month-old male child presented with poor weight gain and recurrent admissions for dehydration. He was the firstborn to non-consanguineous parents with a birth weight of 3000 grams, which was appropriate for his gestational age. The mother was noted to have polyhydramnios in her third trimester. The child was exclusively breastfed till four months of age, following which complementary feeds were started. The mother noticed that the child had increased thirst since four months of age and also passed urine frequently. She also noted that the child’s weight gain was very poor. Dietary counseling was given to her to improve the weight gain of the child during routine vaccination visits. The child had been previously admitted in view of acute diarrheal disease with dehydration. At the time of admission, the child’s weight, height, weight-for-height, head circumference, and mid-upper arm circumference were found to be less than the third percentile in the age-appropriate growth charts. The child had all signs of malnutrition, like loss of the rounded contour of the shoulders, muscle wasting, and intercostal wasting, resulting in prominent ribs and a protuberant abdomen with mild hepatomegaly with no free fluid, as illustrated in Figure 1. He had no peripheral edema or signs of hepatic involvement, like jaundice. 

Developmental history showed a mild delay in psychomotor development. The vitals were normal for his clinical presentation, with normal blood pressure. Polyuria and polydipsia were established during the hospital stay.

Investigations

A blood gas analysis done at the time of admission showed hypochloremic metabolic alkalosis with hyponatremia and hypokalemia (Table 1). Serum electrolytes also confirmed the same. Urinary chloride and potassium were found to be elevated, further clinching the diagnosis of Bartter syndrome. Ultrasonography of the abdomen and renal system was found to be normal. Audiological testing, ophthalmological examination, and echocardiogram were found to be normal. 

**Table 1:** Blood and urine investigations at the time of admission mmol/L, millimoles per liter; mg/dL, milligrams per deciliter

Parameter	Observed value	Reference range
pH	7.739	7.35-7.45
Serum bicarbonate (mmol/L)	23.7	22-26
Serum sodium (mmol/L)	125	135-145
Serum potassium (mmol/L)	2	3.5-5
Serum chloride (mmol/L)	70	98-107
Serum magnesium (mg/dL)	3	1.7-2.2
Urinary chloride (mmol/L)	52	<10

Management

The child was started on oral potassium supplementation, considering the clinical presentation and electrolyte values, and was also encouraged to take a high-salt and potassium diet. Indomethacin was started. Table 2 shows the results of the genetic testing confirming the diagnosis of classic Bartter syndrome type III. The child was followed up after a month, and the repeat blood investigations showed an improvement in his electrolyte values. He also showed an improvement in his growth parameters.

**Table 2:** Whole exome sequencing report This table confirmed the diagnosis of Bartter syndrome (type III).

Pathogenic variant causative of the reported phenotype was detected						
Gene (transcript)	Location	Variant	Zygosity	Disease (OMIM)	Inheritance	Classification
CLCNKB (+) (ENST00000375679.9)	Exons 2-20	chr1:g.(160438 81_16044485) (_16056916_?) del	Heterozygous	Bartter syndrome type 3 (OMIM#607364)	Autosomal recessive	Pathogenic

## Discussion

The clinical presentation is variable in Bartter syndrome, with around 50% of the cases presenting within the first year of life [4]. The symptoms start in infancy with polyuria, polydipsia, vomiting, constipation, and poor growth [5]. Older children may present with recurrent episodes of dehydration and muscle cramps. Nephrocalcinosis, repeated episodes of dehydration, and chronic hypokalemia can cause chronic kidney disease in some patients with Bartter syndrome [6]. Historically, Bartter syndrome type IV has been linked to sensorineural hearing loss [7]. The molecular basis for this condition is a defect in the NKCC in the ascending limb of the loop of Henle. This leads to a loss of sodium, potassium, chloride, and water, which in turn causes secondary hyperaldosteronism. Low serum potassium also stimulates prostaglandin synthesis, which increases the renin and aldosterone synthesis. Laboratory evaluation will show hypokalemic hypochloremic metabolic alkalosis with hyponatremia, hyperreninemia, and hyperaldosteronism. Hypercalciuria may be present in the antenatal form [8].

Devendra and Rowe reported a case of a 23-year-old female with a three-month history of malaise and muscle cramps [9]. Her laboratory investigations revealed hypokalemia, which, despite oral supplementation for four years, remained uncorrected. On further evaluation, urinary chloride was found to be high. This presentation was similar to the present child, but due to the age of the patient, a diagnosis of Gitelman syndrome was made. A similar presentation in younger children would point to a diagnosis of Bartter syndrome [9]. It is very important to differentiate Bartter syndrome from other conditions. In conditions like cystic fibrosis, urinary chloride will be low. Hypomagnesemia is seen more in Gitelman syndrome. There remains considerable overlap between the two conditions. However, the present child had a high urinary chloride and potassium.

The index child had hypokalemic hypochloremic metabolic alkalosis with normal blood pressure. However, Bertulli et al. reported a case of a seven-month-old female child who presented with failure to thrive and was noted to have hypertension [5]. Her laboratory investigations revealed mild hypokalemia with metabolic alkalosis and low renin and aldosterone values. Renal ultrasonography was suggestive of nephrocalcinosis. Due to the association of hypokalemia, nephrocalcinosis, and failure to thrive, a classic Bartter syndrome type III was suspected despite the low renin and aldosterone values. Due to a lack of improvement, the child was evaluated again four months later. An echocardiogram revealed ventricular septal hypertrophy. Hypertension, hypokalemia, and low renin and aldosterone levels were suggestive of a monogenic form of hypertension. Congenital adrenal hyperplasia was ruled out by routine neonatal screening performed during the neonatal period. Normal deoxycorticosterone, corticosterone, 18-hydroxydeoxycorticosterone, and 18-hydroxycortisol levels suggested a diagnosis of apparent mineralocorticoid excess (AME) syndrome. It was confirmed by 24-hour urine steroid metabolome analysis that showed the typical constellation of 11β-hydroxysteroid dehydrogenase type 2 deficiency with an elevated ratio of tetrahydrocortisol and allo-tetrahydrocortisol to tetrahydrocortisone. Although the clinical presentation of AME syndrome may be very similar to Bartter syndrome, this case highlights the importance of thinking of other diagnoses in cases that present with hypokalemic metabolic alkalosis [5]. 

Renal biopsy is usually not needed for diagnosis. The gold standard for diagnosis is molecular analysis [9]. Since there is currently no effective treatment for Bartter syndrome, managing the metabolic and electrolyte abnormalities are the main goals of care. Consequently, potassium retention diuretics and prostaglandin synthesis inhibitors like non-steroidal anti-inflammatory drugs block prostaglandin synthesis by inhibiting the renin-angiotensin-aldosterone system. Hence, they are considered for the treatment of patients with this condition, as is done in the present child with potassium supplementation and indomethacin treatment. The treatment is usually lifelong, and these children must be observed for signs of renal and gastrointestinal toxicity. Tubulointerstitial nephropathy can occur at a later stage [10].

## Conclusions

Children with malnutrition, though fairly common in developing countries, presenting with signs of malnutrition may confound the clinical picture of some rare disorders like Bartter Syndrome. A diagnosis is grossly delayed in children as a number of laboratory tests need to be done to make it. This case report highlights the fact that a diagnosis of Bartter syndrome must be entertained when presented with a case of failure to thrive with hypochloremic metabolic alkalosis and hypokalemia once the initial workup of severe acute malnutrition is unproductive. Early diagnosis will lead to an earlier correction of metabolic abnormalities, which will improve the child's growth. Given that it is an autosomal recessive disorder, a timely diagnosis helps the couple to get counseled prior to planning their next pregnancy.
